# Polymer Concepts in Cellular Function

**DOI:** 10.1002/advs.76934

**Published:** 2026-08-11

**Authors:** Miao Yu, Shikha Dhiman, Dorothee Dormann, Frauke Gräter, Katja Luck, Friederike Schmid, Andreas Walther, Edward A. Lemke

**Affiliations:** ^1^ State Key Laboratory of Metabolism and Regulation in Complex Organisms College of Life Sciences Taikang Center for Life and Medical Sciences Wuhan University Hubei China; ^2^ Department of Chemistry Johannes Gutenberg University Mainz Germany; ^3^ Biocentre II Johannes Gutenberg University Mainz Germany; ^4^ Institute of Molecular Biology (IMB) gGmbH Mainz Germany; ^5^ Max Planck Institute for Polymer Research Mainz Germany; ^6^ Institute of Physics Johannes Gutenberg University Mainz Germany

**Keywords:** amyloid, DNA binding protein, electrostatics, intrinsically disordered protein (IDP), multiscale, neurodegenerative disease, non‐equilibrium, protein interactions, RNA binding protein, transcription

## 1 Polymer Concepts in Cellular Function

For decades, polymer science and molecular biology have developed along largely parallel trajectories. Polymer science has sought to understand how long‐chain molecules give rise to collective properties such as elasticity, viscoelasticity, self‐assembly, phase transitions, and hierarchical organization. Molecular biology, by contrast, has primarily viewed DNA, RNA, and proteins as information carriers, catalysts, and molecular machines that execute genetically encoded programmes. These complementary perspectives have generated extraordinary advances in both disciplines, yet they have rarely been fully integrated.

In recent years, the situation has changed rapidly. A growing body of evidence demonstrates that many cellular functions are not simply governed by the biochemical identity of biomolecules, but by their physical behavior as polymers. DNA, RNA, and proteins are sequence‐defined macromolecules whose length, flexibility, charge distribution, topology, and collective interactions fundamentally determine how cells organize themselves and function in space and time. The recent recognition that phase separation contributes to the formation of membraneless organelles is perhaps the most visible example of this conceptual shift [1, 2], but it is by far not the only example. Chromatin folding, intracellular mechanics, molecular recognition, proteostasis, and signal transduction are just a few of many examples that rely on fundamental principles long familiar to polymer scientists.

At the same time, biology has started to transform polymer science. Living matter represents perhaps the most sophisticated polymer system imaginable. Unlike synthetic polymers, biological macromolecules are chemically heterogeneous, sequence‐defined, hierarchically organized, continuously remodeled, and maintained far from thermodynamic equilibrium through constant energy consumption. They operate within crowded, multicomponent environments where interactions occur simultaneously across molecular, mesoscopic, and cellular length scales. Biological systems not only allow the application of established polymer theories, but also increasingly expose the limitations of these theories and motivate polymer scientists to explore entirely new concepts.

**FIGURE 1:**
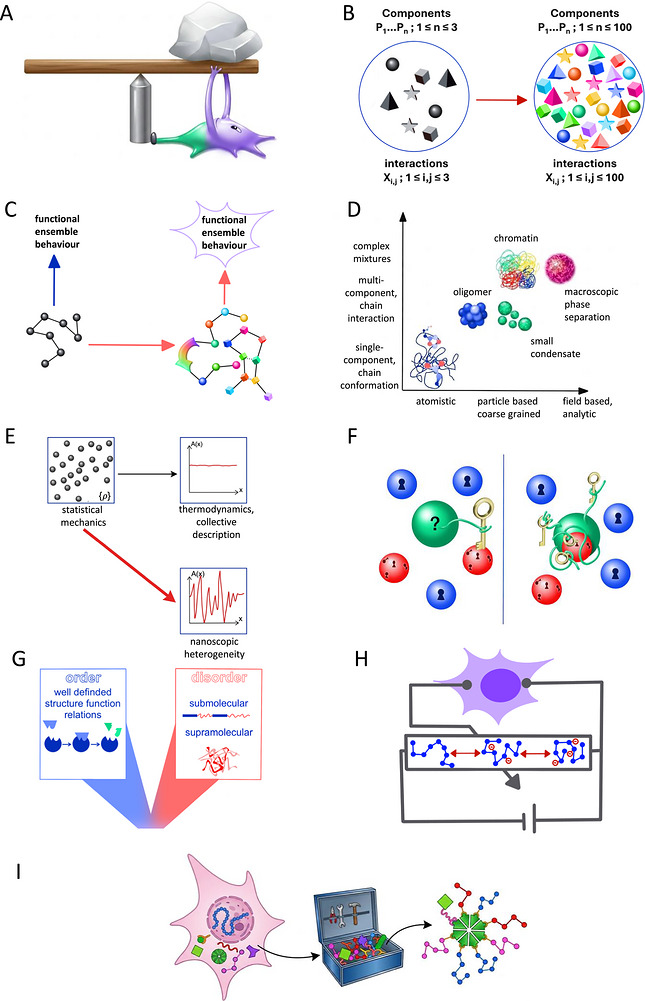
Nine Polymer Concepts relevant to the Polymer Life Sciences. (A) Non‐equilibrium – Explores how continuous energy input drives biological systems away from equilibrium and shapes their behavior. (B) Multi‐component – Studies how highly crowded mixtures with many different biomolecules influence assembly states, phase behavior, and dynamics. (C) System complexity – Extends polymer models to describe the structural and interaction complexity of biomolecules. (D) Multi‐scale – To connect structures and processes across scales. (E) Nanoscopic ensemble behavior – Investigates the unique statistical behavior of very small biological systems where fluctuations are significant. (F) Super‐selectivity – Examines how multivalent molecular interactions enable highly precise and selective recognition. (G) The role of disorder – Studies how structural disorder contributes to the formation of functional order in systems. (H) Tuneable electrostatics – Investigates how dynamic changes in molecular charge regulate interactions and organization. (I) Macromolecular design – Uses precisely engineered polymers and biomaterials to study and control processes.

This reciprocal relationship has given rise to an emerging discipline that we here refer to as *Polymer Life Sciences*: a framework in which polymer science provides the quantitative language for understanding cellular organization, while biological systems inspire the next generation of polymer concepts. Although numerous examples illustrate this convergence, nine concepts appear particularly powerful in bridging polymer science and the life sciences (Figure 1). Together, they describe how living polymers differ fundamentally from their synthetic counterparts while highlighting opportunities for future research.

## 2 Cells Operate Far From Equilibrium

Classical polymer physics is rooted in equilibrium thermodynamics. Living cells are not (Figure 1A).

Virtually every cellular process consumes energy, whether it is through ATP or GTP hydrolysis, powering molecular motors, driving active transport, or sustaining the continuous synthesis and degradation of macromolecules. Biomolecular condensates continuously exchange molecules with their surroundings, chromatin is constantly remodeled, and post‐translational modifications dynamically regulate molecular interactions. Consequently, cells represent dynamic steady states rather than equilibrium structures.

This non‐equilibrium character explains why many biological assemblies defy predictions derived from equilibrium polymer systems. Classical coarsening theory, for example, predicts that small droplets should gradually merge into larger droplets to minimize interfacial energy. Yet many intracellular condensates remain remarkably stable in size despite continuous molecular turnover. Likewise, dynamic post‐translational modifications continually reshape interaction networks without requiring permanent changes in molecular composition. These observations illustrate that understanding living polymers requires extending polymer physics beyond equilibrium statistical mechanics toward active matter and energy‐driven self‐organization.

For example, in this issue, Deck et al. analyze the nucleation kinetics of biomolecular condensates and establish that nucleation is mainly controlled by a single quantity: the interfacial tension between the dilute and dense phases (advs.202522585). Surprisingly, nucleation cannot only yield dense liquid phases within dilute solutions, but also vacuoles, which are dilute voids in the dense phase. If and how such spatial pattern formation impacts biological function is a pressing and yet unresolved question in biology.

## 3 Biological Polymers Exist in Extraordinarily Complex Mixtures

Polymer science has traditionally studied comparatively simple systems—single polymers, binary blends, or ternary mixtures—in carefully controlled environments. By contrast, the interior of a cell contains thousands of macromolecular species whose concentrations continuously change across space and time (Figure 1B).

Crowding is therefore not simply an experimental complication but an intrinsic feature of all cellular systems. Every protein, RNA molecule, or chromatin domain experiences an environment in which countless interactions compete, cooperate, and evolve simultaneously. Such multicomponent behavior profoundly influences phase separation, diffusion, viscoelasticity, and reaction kinetics.

Current theoretical descriptions often simplify these systems dramatically to achieve computational tractability. However, biological function frequently depends precisely on the heterogeneity that such simplifications neglect. Developing theoretical frameworks capable of treating highly multicomponent polymer systems, therefore, represents one of the major challenges at the interface between polymer science and biology.

## 4 Complexity is Functional

Living polymers differ from synthetic polymers not merely because they are more complicated, but because their complexity is functional (Figure 1C).

Proteins combine folded domains with intrinsically disordered regions. RNA forms secondary and tertiary structures while remaining dynamically flexible. Chromatin integrates DNA packed together with proteins at various structural levels. Rather than behaving as uniform chains, biological polymers exhibit sequence‐dependent interactions that change over time and depend on local chemical context.

Capturing such behavior requires polymer theories capable of describing heterogeneous chain architectures, multiblock organization, hierarchical folding, and emergent collective behavior. Importantly, interactions are not only additive. Instead, biological function often arises also from cooperative phenomena in which molecular context determines macroscopic behavior. Biology, therefore, encourages polymer science to move beyond simplified “beads‐on‐a‐string” descriptions toward sequence‐aware, adaptive polymer models.

Almost every publication in this issue that touches on biology studies molecular systems of high complexity. One particularly complex example is transcription regulation in eukaryotes. Klingberg et al. show in this issue that despite this complexity (advs.75924), elegant experiments and simulations can be designed to understand the polymeric nature of DNA and how many transcriptional regulators act together to ultimately control complex processes such as cellular differentiation. A cancer‐relevant example is described in the study by Chong et al. (advs.701606), who show that the oncogenic fusion protein EWS‐FLI1 can form cell cycle‐dependent signaling hubs on chromatin. Some of those structures cannot be resolved using conventional microscopy, making the study particularly challenging and conceptually intriguing from the perspective of nanoscopic ensemble behavior, another polymer concept introduced below. Importantly, these structures respond to specific drug treatments, aligning with the growing belief that targeting the polymeric properties of biopolymers could offer a new therapeutic strategy [3].

As transcription has become a field where polymer concepts find a fertile breeding ground for both application and further development, the review by Quail and Wittmann in this issue shares a more in‐depth look at this field (advs.76934).

Going one step further in the central dogma of molecular biology, translation illustrates how complexity itself can serve as a regulatory principle, enabling cells to adapt their functions. Huang et al. show a particularly intriguing example of how complex RNA‐protein polymer complexes can orchestrate translation and ultimately neural function (advs.202600044).

## 5 Cellular Organization Spans Many Length Scales

Perhaps no feature distinguishes biological polymers more clearly than their extraordinary hierarchy. Atomic interactions determine amino‐acid side chain conformations; proteins assemble into complexes; complexes can organize into condensates; condensates can interact with organelles; organelles coordinate cellular physiology. Similarly, chromatin organization extends continuously from nucleotides to nucleosomes, topologically associated domains, and entire chromosomes (Figure 1D).

No single experimental or computational method can bridge these scales. Instead, genuine understanding requires multiscale approaches that integrate atomistic simulations, coarse‐grained models, continuum theories, and quantitative experiments. Such integration is already transforming soft‐matter physics and is likely to become indispensable for deciphering cellular organization. Rather than viewing coarse‐graining as a necessary approximation, biology challenges us to preserve sequence information, topology, and functional interactions while progressively increasing spatial and temporal scales.

Chen et al. use coarse‐grained simulations to show that reversible cross‐links between polymers (one could call them strong, but reversible stickers) can lead to condensates with exceptionally high water content (advs.202519636). Michels et al. use phase‐field modeling to explain how the formation of strong stickers, e.g., due to the emergence of β‐sheets in originally disordered protein chains, can lead to what is often referred to as “molecular ageing” in the life sciences. This term is often used phenomenologically in biology to describe the hardening of a liquid droplet into a gel state, yet the molecular or polymeric mechanisms underlying this material state transition are understudied and not well understood.

Also in this issue, Mukherjee et al. propose a mechanism of epigenetic inheritance during DNA replication, which is based on polymer‐assisted condensation, and demonstrate its effectiveness by coarse‐grained computer simulations (advs.202512500). Moreover, Tang et al. provide an overview of how computational simulations across different scales combined with machine learning, can help to understand how meters of DNA can be compacted into a small nucleus inside the living cell (advs.74997), in a functional form that does not just store memory, but can be constantly accessed by the transcriptional machinery to keep the cell alive and responsive to its environment.

On a more general level, Shi et al. share with us their view on how AI is already and will make an even bigger difference to drive the field of polymer life science forward from the computational perspective, by offering solutions at nearly all scales, from improving force fields for atomistic simulations all the way to integrative modeling of different data modalities at much larger scales (advs.76023).

## 6 Small Ensembles Can Produce Large Biological Effects

Many studies of biomolecular condensates naturally focus on micron‐sized membraneless organelles. Increasingly, however, attention has shifted toward nanoscopic assemblies that may contain only tens or hundreds of molecules (Figure 1E).

Such small systems challenge conventional statistical mechanics. The thermodynamic limit no longer applies; fluctuations become dominant, and molecular copy numbers themselves may determine biological function. Instead of averaging away stochasticity, cells may exploit it.

Experimentally, these assemblies remain exceptionally difficult to detect and characterize because they lie below the resolution of conventional light microscopy while exhibiting rapid dynamics. Conceptually, they invite polymer science to extend finite‐size statistical mechanics toward biologically relevant systems operating with remarkably small molecular ensembles.

In this issue, Dillenburg et al. present a phosphorylation‐induced switch in the low‐complexity domain of TDP‐43 (advs.76625), a widely studied protein due to its central role in neurodegenerative diseases, such as amyotrophic lateral sclerosis (ALS) and frontotemporal dementia (FTD). When the C‐terminal intrinsically disordered region of the protein is sufficiently phosphorylated, a diblock structure emerges that can microphase‐separate into micelles with a few tens of nanometers in diameter. As neurodegenerative diseases are frequently associated with intrinsically disordered proteins (IDPs) transitioning to amyloid structures, it is important to know the full phase diagram that such biopolymers can populate, as this increases the likelihood of identifying the molecular assembly pathway relevant to disease and, if inhibited, could prevent disease.

Also relevant to protein aggregation diseases, Zhang et al. (advs.202517009) present in this issue that a positively charged domain coupled with an oligomerization domain in a viral protein can tune stress granule formation. Identifying the functional size of the oligomers remains a challenge, as submicroscopic “assemblies” are always harder to study.

As many neurodegenerative diseases are conceptually related, any knowledge gained from TDP‐43 and stress granules can advance our understanding of a broad class of diseases, including ALS, FTD, Alzheimer's, Parkinson's, and Huntington's disease. Thus, in particular in the field of neurodegeneration, it is important to study both levels: the biological specificity of individual proteins, such as TDP‐43, FUS, Tau, synuclein, etc., and the shared principles that govern many IDPs, particularly nucleic acid‐binding IDPs. Thus, not just for those interested in ALS and FTD, Zangrando et al. provide an excellent overview of the multiple faces and functions of TDP‐43 in this special issue (advs.76119).

## 7 Selectivity Emerges From Collective Interactions

Specificity is essential for life. Cells reliably distinguish correct interaction partners despite operating within extraordinarily crowded molecular environments (Figure 1F).

Polymer science provides an elegant explanation through the concept of multivalency and super‐selectivity. Individual molecular interactions may be weak, yet simultaneous engagement of multiple binding sites produces highly nonlinear binding behavior. Small differences in binding site density can therefore generate dramatic differences in molecular recognition.

This principle helps explain how cells achieve robust specificity without relying exclusively on exceptionally strong individual interactions. More broadly, it demonstrates that biological recognition often emerges from polymer interaction networks rather than from isolated molecular events. This concept has also inspired the rational design of synthetic multivalent materials and nanomedicines [4], illustrating once more the strength of reciprocal exchange between biology and polymer science.

Shrestha et al. (advs.202512966) study another pathway for enhancing selectivity: binding‐induced folding. Using elegant temperature jump studies in cells, the authors suggest that nucleic acids first bind to a preexisting folded conformer of the disorder‐rich protein YB1, which then promotes molecular motions to stabilize the bound state of the nucleic acid‐protein complex.

Niblo et al. (advs.75904) used NMR and biochemical studies to elucidate the conformational ensemble of the multidomain ubiquilin (UBQLN) protein family, which contains both disordered regions and folded domains within the same chain. In this issue, their results led them to propose a generalisable mechanism for how tethering different domains together to form multidomain proteins can create highly effective concentrations that favor intramolecular IDR: folded domain interactions in which physicochemical properties, rather than primary sequence, determine interaction specificity.

## 8 Disorder Enables Order

Few developments have altered molecular biology more profoundly than the recognition that intrinsic disorder is not an exception but a widespread design principle (Figure 1G).

From a classical structural biology perspective, disorder appeared synonymous with incomplete folding or with the absence of function. Polymer science, however, has long appreciated that conformational flexibility can generate unique material properties and biological functions. Today, IDPs have emerged as central players in biomolecular condensation, signaling, and molecular organization [5].

Rather than opposing order, disorder frequently enables it. Flexible regions permit dynamic interactions, facilitate multivalency, and allow biological assemblies to remain adaptable while maintaining overall organization. The resulting interplay between structural flexibility and functional specificity represents one of the most fascinating examples of how polymer concepts reshape our understanding of cellular organization.

With about 30% of the human proteome comprising intrinsically disordered regions, there are many examples where disorder‐to‐order concepts can play a role in biology. In this issue, Liu et al. describe how polyglycine‐rich proteins (advs.76630), i.e., a stretch of the simplest amino acid we know and thus a homopolymer, arising from repeat expansions similar to those occurring in Huntington's disease and related inherited neurodegenerative disorders [6], assemble into PolyG fibrils that themselves further organize into higher‐order structures, densely packed ribbons in the cell nucleus.

Disordered proteins are most infamous for their role in neurodegenerative diseases, yet they are also well known in virology, as many viruses try to minimize their genomes and have identified the use of multifunctional IDPs as a means to do so. Gondelaud et al. describe in this issue a special class of IDPs in Hendra viruses where the assembly state from condensates to different fibrillar assemblies is controlled by a redox switch in key cysteine residues (advs.202523465).

## 9 Electrostatics Become Dynamically Programmable

Electrostatic interactions have always occupied a central position in polymer science. Biology adds an additional dimension: cells continuously tune molecular charge (Figure 1H).

Changes in pH, ionic strength, and post‐translational modifications such as phosphorylation dynamically alter the charge of biopolymers and thus the electrostatic interactions between proteins, RNA, and DNA. Proteins behave predominantly as polyampholytes, whereas nucleic acids represent classical polyelectrolytes. Their interactions therefore depend not only on total charge but also on sequence‐dependent charge distribution.

Such tunable electrostatics influence conformational changes, molecular recruitment, phase separation, and mechanical properties of biomolecules. Unlike static synthetic polymers, biological polymers actively regulate their interaction landscapes through tunable electrostatics, providing cells with an elegant control mechanism to rapidly reconfigure molecular organization without altering molecular composition.

The most straightforward way to tune electrostatics in proteins is by phosphorylation, which is controlled by kinases and phosphatases. To give one example, Herrera et al. show in this issue that phosphorylation of the autophagy receptor optineurin (OPTN) induces a ribbon‐like filamentous structure of OPTN (advs.202509927), which can further phase‐separate upon binding to polyubiquitinated cargo and recruit LC3‐positive autophagosomes. Thus, phosphorylation triggers a condensation process that, in turn, enables the selective clearance of dysfunctional proteins from the cell.

In addition to post‐translational modifications, many other mechanisms tune electrostatics in cells. In this issue, Yang et al. show that, counterintuitively, electrostatics can also be tuned even when no charged residues are involved (advs.202524324). Using different IDPs composed solely of uncharged amino acids, they show that micropolarity can emerge in a multiphase system, ultimately leading to ion concentration gradients, such as pH gradients, that can affect biological function. Also in this issue, Phillips et al. use single‐molecule measurements to understand how counterions tune the conformational ensemble of IDPs (advs.202514056), ultimately enabling predictions of their molecular behavior at very low concentrations and supramolecular behavior at very high concentrations.

## 10 Biology Inspires a New Era of Macromolecular Design

The final concept that we discuss here, macromolecular design, goes beyond understanding biology toward engineering it (Figure 1I).

Modern macromolecular design extends well beyond traditional polymer synthesis to include supramolecular chemistry, dynamic covalent systems, peptide engineering, and DNA nanotechnology. These approaches enable precise programming of sequence, topology, connectivity, and hierarchical assembly.

Rather than merely reproducing natural systems, they provide powerful experimental platforms for testing biological hypotheses. Artificial polymers, programmable biomaterials, and synthetic condensates allow researchers to isolate specific molecular variables that are difficult to manipulate within living cells. In this way, macromolecular design becomes not only an engineering discipline but also a fundamental tool for discovery in cell biology.

In this issue, Verwiel et al. (advs.202514449) show how photocrosslinking of synthetic polymers can be used to create systems that bring us one step closer to an artificial cell, as their new “cell‐like architectures” exhibit a DNA‐protective environment.

The review by Shao et al. provides a comprehensive summary of this reciprocal relationship (advs.202600032): how biology can inspire material design, while macromolecular polymer chemistry can provide reductionist yet controllable and programmable chassis for studying complex biological systems.

## 11 Toward a Common Language for Living Matter

The nine concepts (Figure 1) discussed here are not independent. They describe different facets of a single emerging picture in which cellular function arises from collective polymer behavior across multiple spatial, temporal, and organizational scales. Non‐equilibrium dynamics shape multicomponent systems; sequence complexity governs electrostatics, disorder, and multivalency; interactions across scales generate nanoscopic assemblies whose collective properties ultimately determine cellular function.

Importantly, this perspective transforms both the polymer and the life sciences. Polymer science contributes quantitative concepts that explain biological organization, while biology continually challenges polymer theory to incorporate sequence specificity, active matter, adaptive interactions, and hierarchical complexity. Progress, therefore, depends not on transferring knowledge in one direction, but on establishing a genuine dialogue between both disciplines.

In this special issue, Chen and Chilkoti also share their view on how polymer science has emerged as a discipline that now intersects with the life sciences (advs.75240). A full view of this new field would likely require an entire special issue just filled with review articles, but this contribution already gives a superb overview, touching on key aspects from the foundation of polymer science, via the different polymers in nature, going beyond DNA, RNA, and proteins, all the way to the perspective of using polymer concepts to study gene regulatory mechanisms.

In the last few years, many reviews have discussed how considering phase separation, an intrinsic property of any polymer, has changed biology and has had an enormous impact on cell and molecular biology, developmental and tissue biology, all the way to organismal biology. The review by Moschou and Staiger in this special issue complements the existing literature by summarizing the impact of condensate biology on plant science (advs.75297), in particular, how plants employ condensates to integrate temperature, light, redox, and nutrient signals.

The history of science repeatedly demonstrates that conceptual advances occur when previously separate fields begin to share a common language. Polymer concepts have already revolutionized our understanding of synthetic soft matter. Increasingly, they are proving equally fundamental for understanding living matter. We anticipate that the coming decade will see polymer science move from providing useful analogies for biology to becoming one of its central conceptual foundations. Conversely, living systems will increasingly define the frontier at which polymer science develops its next generation of theories, experimental methods, and materials.

The emerging field of **Polymer Life Sciences** is therefore more than an interdisciplinary intersection. It represents a new way of thinking about cellular function—one in which the remarkable complexity of life is understood through the universal principles governing polymers, while the same biological systems redefine what polymer science itself can become.

## Author Contributions

**Miao Yu**: writing – original draft, writing – review and editing, conceptualization. **Frauke Gräter**: writing – original draft, writing – review and editing, conceptualization. **Dorothee Dormann**: writing – original draft, writing – review and editing, conceptualization. **Andreas Walther**: writing – original draft, writing – review and editing, conceptualization. **Friederike Schmid**: writing – original draft, writing – review and editing, conceptualization. **Shikha Dhiman**: writing – original draft, writing – review and editing, conceptualization. **Katja Luck**: writing – original draft, writing – review and editing, conceptualization. **Edward A Lemke**: writing – original draft, writing – review and editing, conceptualization.

## Funding

This work was supported by the Deutsche Forschungsgemeinschaft (DFG, German Research Foundation)‐SFB1551‐“Polymer concepts in Cellular Function” Project No. 464588647.

## Conflicts of Interest

The authors declare no conflicts of interest.

## Data Availability

Data sharing not applicable to this article as no datasets were generated or analyzed during the current study.
